# Validation of Individual-Based Markov-Like Stochastic Process Model of Insect Behavior and a “Virtual Farm” Concept for Enhancement of Site-Specific IPM

**DOI:** 10.3389/fphys.2016.00363

**Published:** 2016-08-23

**Authors:** Slawomir A. Lux, Andrzej Wnuk, Heidrun Vogt, Tim Belien, Andreas Spornberger, Marcin Studnicki

**Affiliations:** ^1^inSilico-IPMKonstancin-Jeziorna, Poland; ^2^Department of Applied Entomology, Warsaw University of Life SciencesWarsaw, Poland; ^3^Julius Kühn-Institut, Federal Research Centre for Cultivated Plants, Institute for Plant Protection in Fruit Crops and ViticultureDossenheim, Germany; ^4^Department of Zoology, pcfruit vzwSint-Truiden, Belgium; ^5^Division of Viticulture and Pomology, University of Natural Resources and Life SciencesVienna, Austria; ^6^Department of Experimental Design and Bioinformatics, Warsaw University of Life SciencesWarsaw, Poland

**Keywords:** *Rhagoletis cerasi*, European cherry fruit fly, virtual farm, agent-based models, site-specific IPM

## Abstract

The paper reports application of a Markov-like stochastic process agent-based model and a “virtual farm” concept for enhancement of site-specific Integrated Pest Management. Conceptually, the model represents a “bottom-up ethological” approach and emulates behavior of the “primary IPM actors”—large cohorts of individual insects—within seasonally changing mosaics of spatiotemporally complex faming landscape, under the challenge of the local IPM actions. Algorithms of the proprietary PESTonFARM model were adjusted to reflect behavior and ecology of *R. cerasi*. Model parametrization was based on compiled published information about *R. cerasi* and the results of auxiliary on-farm experiments. The experiments were conducted on sweet cherry farms located in Austria, Germany, and Belgium. For each farm, a customized model-module was prepared, reflecting its spatiotemporal features. Historical data about pest monitoring, IPM treatments and fruit infestation were used to specify the model assumptions and calibrate it further. Finally, for each of the farms, virtual IPM experiments were simulated and the model-generated results were compared with the results of the real experiments conducted on the same farms. Implications of the findings for broader applicability of the model and the “virtual farm” approach—were discussed.

## Introduction

European regulations stipulating integrated pest management (IPM) (Directive 2009/128/EC^1^), scarcity of robust and economically sound IPM methods and dwindling lists of the approved pesticides (European Commission, DG SANCO, 2013)—combined—render the fresh production increasingly challenging. The problem is particularly acute in spatiotemporally complex medium scale farming systems typical for “ecologically-conscious” non-industrial fruit and vegetable production, where generic IPM protocols are seldom effective without site-specific adaptation. Indeed, the local IPM performance is determined by the local farm topography and traits, which shape the outcome of fine interplay among concurrent processes, where the on-farm dwelling cohorts of individual, independently operating insects are the key causative actors. The latter merits a “bottom-up” and individual-focused “ethological” approach (Lux, 1992, 1994; Lux and Gaggl, 1996), and application of Markov-like processes in individual-based stochastic models (Lux, 1989, 2014; Grimm et al., 2005). Through inclusion of the site-specific spatiotemporal features, such models could serve as a “virtual farm” emulating the key processes determining performance of the local farming system, and offer quantified insights into the mechanisms driving the local IPM performance.

Such individual- and/or agent-based models, enacting complex processes by the actions of their key “virtual actors,” offer unprecedented flexibility and heuristic advantages (Fajardo, 2009). For this reason, the agent-based modeling and the concept of “virtual environmental laboratories” is increasingly applied in studies on complex systems in ecological and evolutionary research (DeAngelis and Mooij, 2005; Jovani and Grimm, 2008; DeAngelis and Grimm, 2014), development of environmental management (Reed et al., 2016) and decision making tools (Grimm et al., 2014), land management and urban planning (Parker et al., 2002; Parker, 2005). Regrettably, in the domain of horticulture and the IPM, the potential of such approaches still remains largely unrecognized and underutilized.

Our objective was to explore the potential of an agent-based “virtual farm” approach for simulation of the local IPM experiments, and “virtual” assessment of the net effects of multiple, concurrent modifications introduced into the local system. The paper reports approaching such task with application of the PESTonFARM model (Lux, 2014), adapted to the cherry growing system and its key pest—the European cherry fruit fly, *Rhagoletis cerasi*. To accomplish this task, our “interim” aim was to assemble and categorize scattered, published and “gray,” knowledge about ecology and behavior of the target pest (*R. cerasi*), and supplement it with auxiliary on-farm observations and experiments. Afterwards, encapsulate the pertinent information into model procedures and parameters, and convert it into an operable tool suitable for the local IPM enhancement. In the model, actions and fate of the virtual individuals enacting the IPM process (*R. cerasi* females) are stochastically determined by the assigned set of behavioral rules and parameters, and modulated by the status of the location (farm sector) of their actual residence. Farm topography and traits are represented by set of grids, with quantified sectors, which values fluctuate during the season, according to plant phenology, IPM treatments etc.

Practical application of the model, as a site-specific IPM optimization tool—was intended from the onset. Alike, converse use of the model for adjustment of the local agro-landscape and designing “pest resilient” farm topographies. Our focus was to obtain assessments for the units relevant to the end-user, such as plots containing various cherry cultivars, IPM treatments, sets of monitoring traps, etc. Although, the model simulates all the processes for each farm (grid) sector, *R. cerasi* typically occurs at very low densities, thus the numbers generated daily for each sector tend to be erratic and of limited practical interest. Our pragmatic goal was to use the model rather for development of typical site-specific IPM tactics, optimized for the local farm topography and the locally prevailing climatic conditions, rather than adjusting the on-farm decisions to the weather fluctuations of a particular season (although such application is also conceivable).

Concise presentation of the agent-based models presents a challenge. They can be characterized by their purpose, description of causative agents, the set of rules and interrelations taken into account, assumptions and estimates of the key parameters, etc. (Grimm and Railsback, 2005). But unlike mathematical models composed of formal, explicit, and easy to scrutinize equations with closed form solutions describing changes in the studied system, the rule-based simulation models are strictly focused on active emulation of interactions among the individual causative actors (agents) and the system (An et al., 2009). The “end result” of the simulation process is neither determined nor programmed, each time it “emerges” *de novo—*generated by the activity of its agents'. Inadequacy of established terminology and universally accepted practice for presentation and testing ecological models (Grimm and Railsback, 2005)—compounds the difficulty even further. Nonetheless, growing popularity of the agent-based modeling fostered advancements in implementation of the presentation standards (Grimm et al., 2006, 2010, 2014; Polhill et al., 2008), and thus the model description largely follows the ODD (overview, design, concepts and details) protocol proposed and updated by Grimm et al. (2006, 2010).

Further to the outline of the model, its parameterization and on-farm validation, a few examples of its potential for IPM enhancement are discussed.

## Methods

### Outline of the model

PESTonFARM (Lux, 2014) is a proprietary, site-specific agent-based model, implemented in the Visual Basic for Applications (VBA) (MS Office for Mac 2011) and fully integrated with the commonly used MS Excel. In the reported study, recently enhanced generic 3.1 version of the PESTonFARM model was used, which can emulate behavior and development of multivoltine pests with multiple, overlapping generations and age-cohorts, operating within seasonally fluctuating mosaics of the local farming landscape, according to the local weather conditions. It reflects farm topography and its key features, emulates host-plant phenology, and behavior of the local pest population during a “virtual” IPM experiment. Upon each run, it generates a unique, but stochastically equivalent set of projections/results presented in the formats resembling real on-farm experiments. All on-farm phenomena are simulated with 1-day temporal resolution. Spatial resolution is determined by pest biology and its estimated daily mobility ranges. The model consists of two main modules: “virtual insect” and “virtual farm.”

*Virtual insect module:* The “virtual insect” module encapsulates relevant information about insect ecology and behavior, and accordingly, determines (in a stochastic sense) behavior of individual “virtual” insects—members of cohorts representing the local pest population. Insect behavior is assumed to resemble a Markov-like process, each behavioral step, event or “decision” of each individual “virtual” insect is fully randomized and stochastically dependent on its age, weather conditions, current status of the sector of its actual residence, and where relevant—also that of the nearby sectors.
*Virtual farm module:* The “virtual farm” module encapsulates relevant information about the particular farm/site to be modeled, and constitutes “virtual environment” which determines development and behavior of the local “virtual insect” population. Various farm aspects are represented by dynamic matrices of quantified square sectors, which values fluctuate daily throughout the season according to the local host phenology, spatiotemporal efficacy profiles of the applied IPM treatments, and the changes imposed by actions of the “virtual insects” themselves.

The key parameters and relations adopted in the model are outlined in Table 1, while their biological background is discussed under the results section. More detail model description, according to the ODD (overview, design, concepts and details) protocol proposed and updated by Grimm et al. (2006, 2010), is provided in the Complementary materials.

**Table 1:** **The main aspects of biology, key processes taken into account, and adopted parameters**.

**Aspect**	**Process/Parameter**	**Adopted values**	**Relation/sub-model**	**Basis/Source**
Adult females	Sex ratio of adults emerging in spring	1:1	Constant	Assumed, based on Łȩski, 1963; Daniel and Grunder, 2012
	Pattern of adult emergence in spring	Staggered, lasting 35–50 days, with 70–90% emerging during peak 14 days	Bell-shaped function adjusted to fit the published data, adjusted to the local farm conditions	Vogt et al., 2010, historic data, aver. temperatures prevailing in spring in each location
	Lifespan (average under optimal conditions and in absence of extrinsic mortality causes)	59 days	Constant	Assumed, based on: Köppler et al., 2008; Moraiti et al., 2012
	Maximum modeled individual lifespan	95 days	Constant	
	Intrinsic age-dependent adult daily mortality risk	Daily average calculated according to cohort age	Gompertz function adjusted to fit published data	
	Extrinsic daily mortality risk caused by complex of on-farm resident predators and natural enemies	3%	Constant (except the areas of pesticide application, with transient suppression of the local natural enemies)	Broadly estimated, based on analysis of historic data
Immature stages	Status of eggs	Fertilized (100%)	Constant	Assumed
	Sex ratio	1:1	Constant	Assumed
	Duration of in-fruit development (from egg to mature larva jumping out from the fruit for pupation)	20–23 days	Constant, adjusted to the locally prevailing temperatures	Assumed based on: Daniel and Grunder, 2012; Łȩski, 1963; Vogt et al., 2010
	Combined mortality from egg till the adult emerging next spring	92%	Constant	
Fecundity	Mating status of mature females	Mated (100%)	Constant	Assumed
	Potential lifetime fecundity (under optimum conditions, unlimited availability of food and suitable fruit, absence of extrinsic mortality causes)	365 eggs/female	Constant	Assumed based on: Köppler et al., 2008; Moraiti et al., 2012
	Batch size	1 egg/fruit	Constant	Assumed based on: Daniel and Grunder, 2012 Based on: Köppler et al., 2008; Moraiti et al., 2012
	Intrinsic age-dependent daily fecundity	Range: 0–10, daily average calculated according to cohort age, individual values generated assuming normal distribution	Asymmetric bell-shaped function adjusted to fit published data	
Mobility	Area covered during a single local exploration errand	100 sqm	Constant	Assumed based on: preliminary on-farm observations, and Böckmann et al., 2012, 2014; Daniel and Grunder, 2012; Daniel and Baker, 2013
	Farm sector size	100 sqm (10 × 10 m)	Constant	Adopted to fit insect's local exploration range
	IN/OUT balance between emigration from the farm and immigration from the neighborhood	1:1	Constant	Assumed for all scenarios presented in the paper
	Micro-migration	Range 30–300 m, potential daily average and SD calculated according to cohort age, individual values generated based on average and SD	Custom-build age-dependent functions	Based on mark-recapture experiments, and Boller, 1969; Łȩski, 1963; Wiesmann, 1935; Vogt et al., 2010
**Aspect**	**Process/Parameter**	**Adopted values**	**Assumed relation/sub-model**	**Basis/Source**
Cherry phenology, fruit suitability and infestation	Cherry phenology	Flowering time, beginning of fruit suitability, harvest	Cultivar-specific	Recorded on farm
	Fruit suitability for oviposition	From the point of hue change (green to yellowish-green) till harvest	Typically: 31–44% of the average flowering-to-harvest period	Vogt et al., 2010, on-farm records of cultivar phenology
	Daily fruit attractiveness and suitability for larval development	Ranging from 0 to 100%, maximum same for all cherry cultivars	Asymmetric bell-shaped function, max. 1/3 of the fruit suitability period	Assumed, function adjusted to fit on-farm recorded cultivar phenology
	Post-infestation fruit recovery time (if egg or young larva was killed e.g., by a systemic pesticide)	5 days, counted from the day of the egg deposition	Constant	Estimated, based on preliminary observations
	Pre-harvest “concealed” fruit injury, when *de facto* infested fruit still appears unblemished to the consumer.	4 days, counted from the day of the egg deposition	Constant	Recorded on farm
Niche utilization	Fruit infestation [%]	Actual for each sector	Custom build functions, according to type of behavior, with minor impact at low to moderate infestation level	Estimated, based on preliminary observations and analysis of previous (historic) trapping data
	Local population density	Actual for each sector		
**Aspect**	**Process/Parameter**	**Adopted values**	**Relation/sub-model**	**Basis/Source**
Monitoring with Rebel traps	The effective trapping area surrounding Rebel trap	100 sqm (10 × 10 m)	Constant	Estimated, based on preliminary observations and analysis of previous (historic) trapping data
	Responsiveness of females to Rebel trap	Age dependent, ranging from the initial 80%, to 100% at peak, and declining to 40% afterwards	Asymmetric bell-shaped function adjusted to fit the assumed thresholds	
	Average daily trapping risk for a new Rebel trap, within the range of its activity	5%	Constant	
	Daily decline in trap original trap efficacy, due to dust etc.	1%	Constant	
Pesticide application	Only the insects present in or entering pesticide zone were deemed exposed to additional mortality risks	Daily mortality risk dependent of estimated residual pesticide effectiveness	Custom-build functions	Estimated, based on: Lazić et al., 2014, and producer's application guidelines
	In the areas of pesticide application, transient suppression of the locally resident natural enemies occurs	Daily recovery rate of the natural enemies dependent of estimated residual pesticide effectiveness	Custom-build functions	
Weather impact	Temperature threshold for mating	15°C	Constant	Daniel and Grunder, 2012; Katsoyannos, 1979
	Temperature threshold for oviposition	16°C	Constant	Boller, 1966; Daniel and Grunder, 2012
	Conditions for explorative activity	No rain, temperature > 13°C, wind < 12 m/s, sunshine > 100 W/m^2^	Custom-build functions	Estimated, based on observations, and Boller, 1966; Daniel and Grunder, 2012; Katsoyannos, 1979

### Identification of the relevant aspects to *R. cerasi* biology, and model adaptation

The generic model, PESTonFARM v. 3.1, was adapted to reflect the key aspects of *R. cerasi* biology. A catalog of the relevant behaviors and on-farm processes was identified based on review of the published information and gray literature, extrapolations from closely related species, compiling experiences of the authors', and the results of our *ad-hoc* on-farm observations and auxiliary experiments. Raw results of the “historic” experiments conducted on-farm in the past (JKI, BOKU, PC-Fruit) were used for calibration of the model algorithms, in particular parameters of its “virtual insect” module. Afterwards, the model was “locked” and subject to on-farm validation without any further adjustments to its internal parameters.

The main biology aspects identified and used for the model adaptation, assumptions and the basis for estimation of the key parameters—are presented in the “Results” section, while the specific model assumptions, derived processes and adopted parameters are presented in Table 1.

### Model validation

Model validation was conducted on three sweet cherry farms located in Germany, Austria and Belgium. For each farm, a customized “virtual farm” module was prepared, reflecting its key spatiotemporal features, which was used to simulate the IPM experiments “really” conducted on the farm, and also additional “hypothetic” IPM scenarios. For each IPM scenario modeled, simulation was replicated five times, and its average results were compared with the experimental data collected on the real farms. All five replicates were made with the same initialization settings, describing the experimental assumptions, status and spatiotemporal properties of the respective farms and IPM treatments. Before any comparisons with the experimental results, homogeneity of the simulated replicates was tested and confirmed.

Capacity of the model to reproduce the mark-recapture experiment (dynamics of the re-capture process and patterns of relocations among farm structures) for each pest age category, and the field results (spatial and temporal patterns of trap catches during pest monitoring, fruit infestation patterns, effects of the local pesticide application etc.)—was treated as an evidence of the correct model calibration and validity.

### On-farm experiments

#### Experimental locations

**Figure 1:**
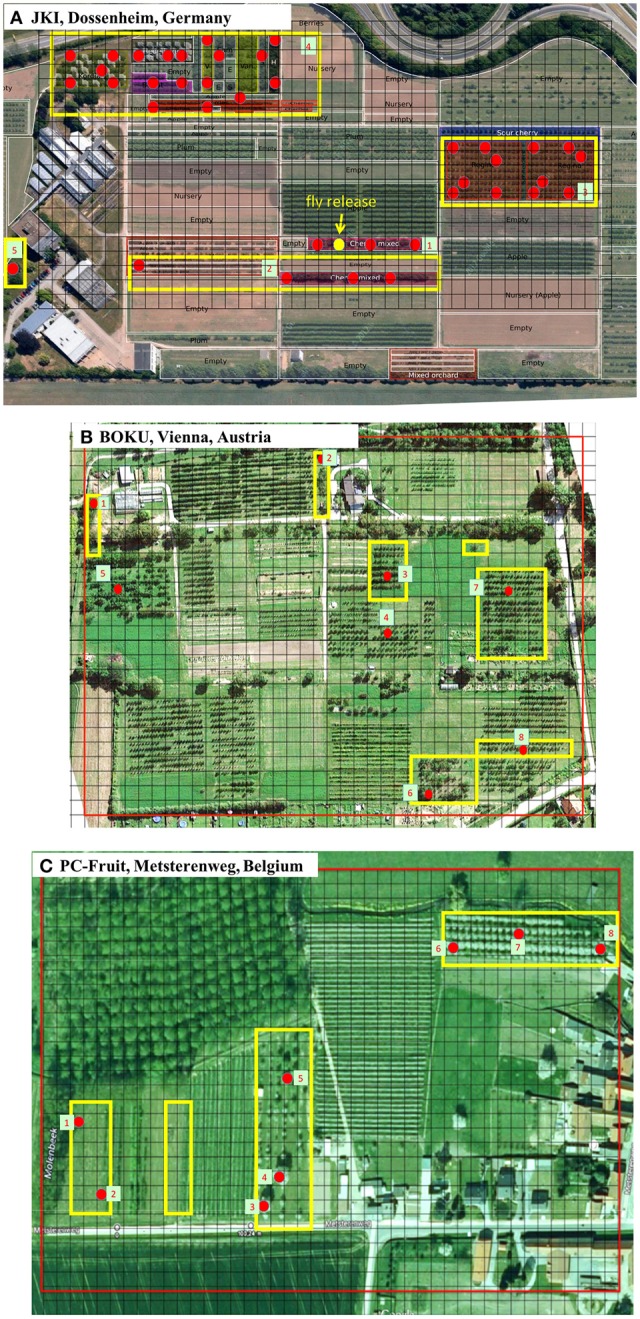
**Outline of the experimental farm topography**. Red frames: area selected for modeling, Yellow frames: plots containing sweet cherry trees, Red dots: position of the Rebel traps, Grid: farm sectors equivalent to 10 × 10 m on the ground, Background map data: Google Maps.

The presented research were conducted on the following locations: (1) Julius Kuhn Institute (JKI) farm, Dossenhem, Germany (N 49°26′ 56.1063″; E 8°38′ 22.3636″; 115 m altitude), (2) University of Natural Resources and Life Sciences (BOKU) farm, Vienna, Austria, (N 48°17′19″; E 16°25′43″; 162 m altitude), (3) Proefcentrum Fruitteelt VZW (PC-Fruit), Metsterenweg, Belgium, (N 50°50′34″; E 5°10′24″; (4) WULS-SGGW campus, Warsaw, Poland, (N 52°10′03″; E 21°02′44″; 101 m altitude). The locations are referred to as follows: JKI farm, BOKU farm, PC-Fruit farm, and WULS campus, respectively. The overall topography of the farms selected for modeling, arrangement of the plots containing sweet cherry trees and positions of the monitoring traps (Rebel) are presented on Figure 1.

#### Insect material

Insect collections and treatment followed methodology of Köppler et al. (2010). Larvae of *R. cerasi* were obtained from field-infested cultivated sweet cherries collected in the experimental orchards of JKI farm and from wild cherry trees growing at WULS campus. The collected larvae pupated within a few hours, and the pupae were kept at room temperature for ca. 8 weeks. Afterwards, to facilitate obligatory diapause, the pupae were stored for at least 195 days at 4°C, 65% ± 5 RH, and kept as a “cold stock” in such conditions for up to 11 months.

To initiate post-diapause development and eclosion, the required numbers of pupae were randomly selected from the “cold stock” and transferred for 25–30 days to climatic chambers set at 25/18°C day/night, respectively, 65 ± 5% RH and L16/D8 photoperiod. After eclosion, both male and female flies were transferred to larger BugDorm cages (30 × 30 × 30 cm) and kept in the same ambient conditions. Males and females were either mixed or kept separately, according to experimental needs. Each cage was supplied with water and food sources (dry yeasts mixed with sucrose, 1:4), offered on Petri dishes *ad libitum*. Water was offered on a wet piece of sponge, with one end submerged in a 100 ml container filled with water, and the other end protruding through a hole in the container's lid. Water was changed daily, while food every third day. Whenever, flies of various age categories were required concurrently, batches of pupae were treated as above in a staggered manner, accordingly. Age classes were assembled by random selection of active and “healthy looking” flies with undamaged wings from the respective batches.

#### On-farm dispersal

The Mark-Recapture experiment was conducted at JKI, using insects from JKI stock. Cohorts of 5-, 14-, and 28-day-old females (ca. 300 females each) were concurrently released from the same point. Each individual was marked with a spot (ca. 1 mm diam.) painted on the thorax, different color for each age category. To intercept the flies relocating within the farm, an array of 38 Rebel traps was established. The traps were checked daily, and numbers of the marked flies trapped were recorded. The experiment lasted 15 days until no more marked flies were caught. Farm topography is presented on Figure 1A. Each of the five re-capture zones contained plots of fruiting sweet cherries and a set of Rebel traps. The release zone (1) consisted of a single elongated plot of fruiting cherry trees (mixed cultivars) with three Rebel traps. Adjacent zone (2) comprised parallel plots with mixed cultivars and four traps, separated from the release zone by only a band (ca. 20 m wide) of empty field. Zone three, consisted of a large plot of Regina cultivar with 12 traps, with its center 147 m and its closest edge 90 m apart from the release point, separated from the release zone by a plot of apple trees. Zone four was the largest, contained several cherry plots with various cultivars and 19 traps, its center was 191 apart from the release zone. The last, fifth zone comprised only two large “wild” cherry trees, growing 238 m from the release point. The remainder of the farm contained a number of plots with apple or plum trees, spaced by several empty fields.

#### Potential impact of parasitoids on *R. cerasi* population

Two samples of pupae, originating from the JKI and WULS stocks, were used. Both samples originated from plots with a history of no recent pesticide application. After 165 days of diapause and cold storage, followed by 21 days of post-diapause development in climatic chambers, numbers of parasitoids emerging from the pupae were recorded daily. The parasitoids were identified by Prof. Kees van Achterberg, Naturalis Biodiversity Center, Leiden, The Netherlands.

#### IPM experiments

The IPM experiments were conducted on two farms (BOKU, & PC-Fruit farms), each containing several plots of various sweet cherry cultivars, various non-host fruit trees and plots with no trees (Figures 1B,C). The farms varied substantially in terms of latitudinal position and climatic conditions, size, spatial arrangement, tree structure, cultivar composition, and management practice. On each farm, usual IPM operations were conducted, supplemented by pest monitoring. On each farm, the locally established populations of *R. cerasi* were present, greatly varying in population density among the farms. Each on-farm experiment comprised the following steps:

- *Spatial farm characterization*: A satellite picture of each farm was used for preliminary farm characterization. On each farm, a grid of square sectors (equivalent to 10 × 10 m on the ground) was superimposed and a rectangular “modeling” area (marked by red frame on the farm map) was selected, ca. 9 and 12 hectares, BOKU and PC-Fruit, respectively. Every grid sector was individually characterized by: presence or absence of tree canopies, their average diameter, degree (%) of land coverage by the canopy, dominant species of non-host trees or cultivar of host trees, row direction etc. Afterwards, detailed surveys were conducted on each farm to verify the information “on-the-ground”. Based on the collected information, for each farm, a customized farm-representing module was prepared, describing the key spatiotemporal farm features such as crop phenology and distribution patterns, tree canopy size, coverage and row directions, non-host plot arrangements, etc. For the modeling process, all the on-farm present cherry cultivars were categorized into four phenological groups: early, medium, late and very late, and for each group a representative cultivar was assigned from among those locally present on-farm. For each grid sector containing sweet cherry trees, appropriate representative cultivar was assigned according to the overall cultivar composition on the plot. Estimation of the initial pest population in each farm sector (expected adult emergence from the soil in spring) was made based on historic pest monitoring records, cultivar category, tree size, distribution, and canopy status etc.
- *Phenology of fruit development:* The key points in host phenology, such as flowering, onset of fruit susceptibility to *R. cerasi* infestation and fruit maturity/harvest time were recorded for the main on-farm present sweet cherry cultivars. The points were defined as follows: time of flowering—when 30% of flowers open, onset of fruit susceptibility—fruit color change from dark green to yellowish—green (30% of fruits), fruit maturity/harvest time—actual harvest dates. The fruit susceptibility period was deemed to last from the time of fruit color change till harvest.
- *Pest monitoring:* At the onset of 2015 season, on each farm, 7–8 Rebel traps were set for pest monitoring. Position of the Rebel traps on each farm is shown on Figure 1. The traps were checked at various, ca. weekly, time intervals, following the usual management practice. During each trap check, all the flies trapped were removed and their numbers recorded. When males and females were not separated, 1:1 sex ratio was assumed (Ozdem and Kilincer, 2008), and half of the catch (to represent only females) was used for simulations.
- *IPM treatments:* For each farm, records were maintained about fruit development, fruit infestation at harvest, average weight of a single ripened fruit, approximate crop yield, pest management actions such as pest control sprays, type and dose of pesticide, time and area of application, etc. To assess fruit infestation, 100 fruits were randomly collected at the time of harvest and dissected. Crop yield was based on farm records, or on grower's assessment. Average weight of a single fruit was estimated by weighting ca. 100 mature fruits, randomly collected at harvest.
- *Weather data:* For each farm, records of daily maximum, average and minimum temperatures (recorded at 2 m above the ground), rain, wind speed (at mid-day), and sunshine (radiation) intensity were used.

### Statistical analysis

The experimental, on-farm collected data were compared with the model-generated results of analogous “virtual” experiments. Simulation of each “virtual” experiment was replicated 5 times. Before comparisons, homogeneity of the model-generated results was verified using the chi square χ^2^ test, and in all cases, confirmed. The overall patterns of the experimental trap catches, both for the mark recapture experiment and on-farm pest monitoring, were compared with the model generated results using the chi square χ^2^ test of goodness-of-fit. Due to small numbers of events, Monte Carlo simulations to corrected *p*-value from chi-square test was used. All the statistical analysis were carried out using R software version 3.1.3 (R Development Core Team, 2015). In addition, a simplified process control test was used, and it was assumed that the process is acceptably controlled (simulated), if the experimental points fall within the 3-sigma control limits for the respective simulated points, and relative distribution of the experimental and simulated points approximates random - no sequences of “+ + + ” or “− − −” longer than 6 (*p* < 1%).

## Results

### Identification of the biology aspects relevant to *R. cerasi*, and model adaptation

The aspects relevant to *R. cerasi* biology and model adaptation were identified based on literature review, the author's experience, and our auxiliary experiments and observations. The result of the review, the aspects selected for modeling, and their relevance to the model adaptation are outlined below, while the derived model processes and parameters were summarized in Table 1.

#### General traits

The European cherry fruit fly is a univoltine pest, and the females, which emerge from the soil in spring, reach maturity within 5–14 days, and deposit eggs into cherry fruits causing crop damage (Łȩski, 1963; Daniel and Grunder, 2012). Under typical field conditions, sex ratio of the adults emerging in spring approximates 1:1, and the majority of females are fertilized (Daniel and Grunder, 2012). Therefore, only females of *R. cerasi* were considered in the simulations.

The process of adult emergence is not synchronized, typically, it lasts 35–50 days, with a culmination in the middle of this period, when 70–90% of individuals emerge during the peak 14 days Vogt et al. (2010). Accordingly, such staggered emergence was modeled, with parameters adjusted to the local farm conditions. Effectively, this phenomenon generates 35–50 consecutive and overlapping age-sub-cohorts, which were modeled separately.

The overall mortality per generation, from the egg till the adult stage emerging from the soil next spring, fluctuates seasonally, usually within the range of 85–98% (Łȩski, 1963; Vogt et al., 2010; Daniel and Grunder, 2012). Female longevity and fecundity varies among locations and seasons, but under the optimal conditions of unlimited food and fruit supply and the absence of any extrinsic mortality causes, the adult lifespan fluctuates around 60 days, with its maximum exceeding 90 days, while gross fecundity may reach 365 eggs/female, with average daily fecundity rates ranging from 0 to 10 eggs/female, according to female age (Köppler et al., 2008; Moraiti et al., 2012). Model parameters were adjusted accordingly, tuned to fit the published data for JKI strain (Köppler et al., 2008). In the absence of specific information, parameters of the JKI strain were deemed to approximate that of the other strains simulated in the reported study (BOKU and PC-Fruit).

#### Insect mobility

The European cherry fruit fly is known for its intimate association with the host tree and its overall mobility largely restricted to the local canopy and its close neighborhood (Böckmann et al., 2012, 2014; Daniel and Grunder, 2012; Daniel and Baker, 2013). Based on our preliminary on-farm observations, the average area covered during a single local exploration errand was estimated at 100 sqm, which was used to determine the basic sector size (10 × 10 m) for all the arrays representing various aspects of the farm, and consequently—the spatial resolution of the whole simulation process. Earlier findings (Wiesmann, 1935; Łȩski, 1963; Boller, 1969), and results of mark-recapture experiments conducted at JKI (Vogt et al., 2010) indicate that occasional micro-migration might exceed a few hundred meters. This was confirmed by our mark-recapture experiment, reported below, which revealed also substantial age-dependant differentiation in several aspects of female mobility, such as varied propensity to undertake the local exploration or on-farm movements, divergent spatiotemporal patterns of the on-farm dispersion etc. These results were taken into account in the process of adjusting the algorithms and parameters of the “virtual” insect mobility module. The possibility of occasional, temporal or permanent, out-of-the-farm migration of some individuals and/or arrival of a number of newcomers from the neighborhood—were also taken into account. Since the experimental farms were located within a similar landscape, a balanced scenario was assumed (in/out migration = 1). Furthermore, insect mobility was assumed to be modulated by the local niche conditions—be enhanced by decreased site attractiveness and/or excessive local pest density or fruit infestation.

#### Tree canopy

Tree canopy constitutes the primary environment for the adult stages of *R. cerasi*, providing shelter and the key attributes required for reproduction. Frugivorous fruit flies, including *R. cerasi*, are known to respond from a distance to visual signals and tree canopy, and adjust their within-canopy behavior according to its size and structure (Prokopy, 1968; Boller, 1969; Katsoyannos et al., 1986; Prokopy et al., 1987; Stadler and Schoni, 1991; Senger et al., 2009; Daniel and Grunder, 2012). Chemical cues, emanating from canopy of various host and non-host trees also play, usually attractive, roles (Lux unpublished). Our on-farm observations indicated further, that apart from the individual tree size, aspects of canopy macro-structure, such as uniformity of tree distribution (tree blocks vs. patchy or scattered) or training the canopy into regular rows, row direction and depth in relation to the open transects, etc.—all play a role in shaping patterns of insect relocations. Consequently, such moderating effects were incorporated into the model.

#### Fruit development and suitability

The European cherry fruit fly is an oligophagous pest, and the presence of cherry fruits at the right stage of development is prerequisite to oviposition. Thus, the “basic” attractiveness of fruitless host tree canopy was assumed to fluctuate at moderate level (ca. 30% of the respective maximum) and substantially increase when the fruit suitable for oviposition becomes present. Details of the fruit development and concomitant physio-chemical changes, especially during its intense growth and early maturation stages, are still little understood (McAtee et al., 2013), and even less so, the corresponding evolution of the fruit sensory qualities and its attractiveness to the insect. Typically, the oviposition starts at mid-late stages of fruit growth, marked by color change from dark green to yellowish-green, culminates at late-growth or early fruit maturation stages and gradually declines until ripening or harvest (Vogt et al., 2010; Daniel and Grunder, 2012). The cultivar-specific periods of fruit suitability were established based on our on-farm records made for the selected cherry cultivars (Table 2). In the absence of specific data, the fruit suitability periods were estimated at 31 or 44% of the average flowering-to-harvest period typical for the cultivar, early or late, respectively, calculated backwards from the harvest time. The latter was based on our results (Table 2) and findings of Schumann et al. (2014) about seasonal dynamics of fruit development, in particular—relation between rapid acceleration in the increase of fruit mass and hue change.

**Table 2:** On-farm (PC-Fruit) recorded “Fruit suitability windows” for ***R. cerasi*** oviposition in various sweet cherry cultivars^*^.

**Sweet cherry cultivar**	**Average F–Y period (days)**	**Average FSW (Y–H period)**		**Average harvest date**
		**(days)**	**(% of the F–H period)**	
**IDENTIFIED SWEET CHERRY CULTIVARS**				
Hertford	59	23	28%	7th July
Kordia	50	29	37%	9th July
Karina	62	19	23%	10th July
Grace Star	54	26	33%	10th July
Lapins	61	30	33%	14th July
Regina	53	40	43%	27the July
Sylvia	50	44	47%	28th July
Sweetheart	59	45	43%	29th July
**UNKNOWN OLD SWEET CHERRY CULTIVARS**				
U1	–	45	–	22nd July
U2	–	45	–	22nd July
U3	–	45	–	22nd July
U4	–	41	–	25th July

* *FSW, fruit suitability window; F, flowering time; Y, fruit colour change from green to yellowish-green; H, harvest time*.

Conceivably, various cherry cultivars may differ in their overall attractiveness and capacity to stimulate oviposition, and their suitability as hosts—capacity to support successful egg-to-larva development. For example, production of hard tissue secluding the eggs and thus reducing their development was reported for some cherry cultivars, such as Schattenmorelle (Thiem, 1954). Although, the model has in-built provision to cater for such phenomena, in the absence of relevant information, the peak attractiveness and the overall host-suitability were assumed equal for all the cultivars. It was assumed further that female intrinsic oviposition propensity be modulated by the status of the sector of her actual presence—enhanced by increase in the overall attractiveness of the local niche and abundance of suitable fruit, and decreased by excessive density of the local pest population or niche exploitation (fruit infestation).

#### Fruit infestation

The European cherry fruit fly is known to utilize epideictic pheromone, deposited on the fruit by the egg-laying female immediately after oviposition to prevent repeated utilization of the same fruit and thus reduce the risk of intra-specific larval competition (Katsoyannos, 1975). This mechanism does not prevent occasional multiple fruit infestations at higher pest pressure, but because in most of the emulated scenarios the fruit infestation was much below 100%, it was assumed that all the eggs were always laid singly (1 egg/fruit). The “in-the-fruit” development time varies depending on temperature and fruit stage (Łȩski, 1963; Vogt et al., 2010; Daniel and Grunder, 2012), and for the locations under the study was assumed to last 20–23 days. Based on our preliminary observations, to enhance model realism, a 5-day post-infestation fruit recovery time, counted from the day of the egg deposition, was assumed for the instances when an egg or young larva died soon after (e.g., due to application of a systemic pesticide), and the initial fruit injury healed without discernible or disqualifying post-infestation symptoms.

#### Fruit harvest

In the absence of systemic pesticide application, harvest is a major factor reducing in-fruit-residing immature population and its carry-over to the next season (Daniel and Grunder, 2012). Therefore, harvest accuracy was estimated for each plot, and the larvae which failed to complete their “in-the-fruit” development period by the harvest time, were considered dead if the fruit was harvested, or assumed completing their development into a pupa if resident in a fruit left on the tree after harvest. Furthermore, because the harvested sweet cherries are typically consumed or processed without delay, additional 4-day period of “concealed” injury was assumed for the instances of just pre-harvest infestation, when *de facto* infested fruit still appears unblemished to the consumer. In all such cases, the eggs were counted toward the overall fecundity, but not to the next generation, and the fruits were treated as “un-infested”.

#### Natural enemies

Based on the analysis of historic data, for all sweet cherry cultivars, the average extrinsic adult mortality risk, due to the on-farm resident natural enemies and pathogens was estimated at 3% daily. Although, seasonal fluctuation is very likely, due to lack of specific data, this effect was assumed constant throughout the season. In our survey, out of 195 individuals of *Psyttalia carinata* (formerly *P. ragoleticola*) recovered, all originated from the larvae collected from wild cherry trees abandoned at WULS campus (34.39% parasitation rate), and none from the cultivated, unprotected cherries. Thus, the impact of larval parasitoids on the immature stages was assumed negligible, even in the absence of pesticide treatments.

#### Rebel traps

Our experience suggests that the number of insects caught by a particular trap depends not only on the general population density, but also on the specific properties of the spot where the trap is located. Individual exposure to the trapping risk is not uniform on-farm, and strictly depends on its position relative to the trap, thus a patchy pattern of the trapping risk was assumed, mirroring trap distribution. The effective trapping range was estimated at 100 sqm, and only the insects present in such area were deemed exposed (in a stochastic sense) to the trapping risk. Implementation of such mechanism ensures that the phenomenon of gradual catch reduction, caused by temporary out-trapping the locally resident flies, was also emulated. Although modulation of the trap attractiveness, caused by seasonal changes in background canopy hue appears likely, due to lack of relevant data—no such effect was included. Based on the results of our mark-recapture experiment, variation in female responsiveness to the trap was assumed, from 80% after emergence, 100% at the peak of reproductive activity, and gradual decline to ca. 40% when 4–6 weeks-old. Because glue-covered traps, exposed in the field for extended periods (ca. 2 months in our study), gradually lose trapping efficiency due to build-up of dust, debris and non-target organisms, for a new Rebel trap, the initial daily trapping risk was estimated at 5%, with a 1% daily decline.

#### Pesticide application

The insects present in or entering pesticide application zone were deemed exposed to additional mortality risks. The date and area of each application were taken into account, along with the estimated temporal profiles of its residues (Lazić et al., 2014) translated into mortality rates of adult flies, and whenever relevant, immature stages developing in the fruit. In such the areas, mortality risk due to the local natural enemies was temporarily reduced to reflect patterns of the pesticide-imposed transient suppression. Specific border effects on the pace and pattern of predator recovery, relative to the size and shape of the area of pesticide application, were also taken into account.

#### Role of weather conditions

The local weather conditions determine behavior of *R. cerasi* and, at more extreme spells, impose mortality risks. The flies are active during warm, calm and sunny days, with temperature above 15°C required for mating (Katsoyannos, 1979; Daniel and Grunder, 2012), and above 16°C for oviposition (Boller, 1966; Daniel and Grunder, 2012). Our observations also confirmed that fly mobility and explorative activity is reduced during cloudy days, especially with some rain or wind. Bad weather spells, with severe rains, especially when combined with strong winds, were assumed to increase fly mortality, according to weather severity and sheltering capacity of local tree canopy.

### Model validation

The validation process consisted of two major steps, simulation of (1) the mark-recapture experiment to validate the model's “insect mobility module” and (2) the IPM experiments conducted on two sweet cherry farms (one in Austria and one in Belgium) to validate the “virtual farm” concept.

#### Simulation of the mark-recapture experiment

The “insect mobility module” is the key model component, which determines on-farm movements of the “virtual” insects. It was evaluated against the mark-recapture experiment, conducted on JKI farm (shown on Figure 1), located in Dossenheim, Germany. The model was set to emulate distribution and the recapture process for each of the three female age categories (5-, 14-, and 28-days-old). The model-generated results were compared with that obtained on-farm. Regardless of the age category, the flies released into the open field remained alive and could be re-trapped during at least 2 weeks post release. In general, out of over 1000 individually marked flies, ca. 6% (64 flies) were trapped. Out of over 300 young (5-days-old) females released—30 individuals (8.7%) were re-trapped, most of them in the release zone (83%), and the remaining 10 and 7% in the zones 2 and 3, respectively. Mature females (14-days-old) were re-trapped at nearly the same rate (8.8%), but relatively fewer in the release zone (55%) and more in the zones 2, 3, and 4 (17%, 10%, 17%, respectively). For the old females (28-days-old), the overall re-capture rate was much lower (1.6%), and thus the catches were much more erratic. Nevertheless, almost half of the re-trapped females were caught in the distant zones (3 & 4), and interestingly, none in the adjacent one (zone 2).

**Figure 2:**
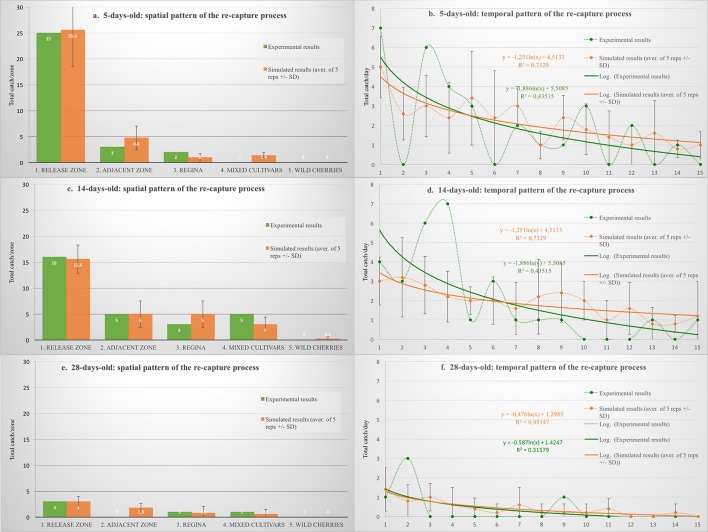
**The mark-recapture experiment: comparison between experimental vs. model-generated age-dependent recapture patterns**.

The model-generated results of the “virtual” mark-recapture experiment largely mirrored that obtained on-farm, both in terms of their spatial and temporal patterns (Figure 2). For each of the three female age categories, no significant discrepancy between the respective simulated and experimental results was detected (Table 3). Furthermore, barring the exception of one point only (Figure 2D, day 4), all other experimental points fell within the 3-sigma control limits of the respective simulated data points, and the relative deviations of the simulated and experimental points did not depart from random (no sequences of “+ + +” or “− − −” longer than six).

**Table 3:** **Comparison between re-capture patterns: experimental vs. model-generated**.

**Source data**	**Chi-square**	**df**	***p*-value**
Figure 2A. 5-day-old: spatial pattern	2.0350	4	0.5681
Figure 2B. 5-day-old: temporal pattern	12.0896	14	0.5991
Figure 2C. 14-day-old: spatial pattern	1.2044	4	0.7964
Figure 2D. 14-day-old: temporal pattern	10.9554	14	0.7567
Figure 2E. 28-day-old: spatial pattern	1.8145	3	0.6118
Figure 2F. 28-day-old: temporal pattern	5.6095	11	0.8961

Admittedly, due to laborious nature of the mark-recapture experiment, the numbers of the experimental data points were low, which prevented establishment of precise “reference” distribution patterns, and thus more rigorous model calibration. Provision of over 1000 individually marked flies, ready for release the same day and representing the three, broadly different age cohorts, presented a challenge. Several thousand of difficult to obtain *R. cerasi* pupae had to be used for this purpose, and substantial increase of these numbers was not feasible. Nonetheless, the results revealed earlier unknown, age-related differences in *R. cerasi* distribution propensities, distances and patterns, which were broadly replicated by the model.

#### Simulation of the on-farm IPM experiments

The IPM experiments were conducted on BOKU farm located near Vienna in Austria and PC-Fruit farm located in Metsterenweg in Belgium. Each farm contained a number of plots with sweet cherry cultivars of varying phenology, some old abandoned cherry trees, plots with non-host fruits trees, wild trees and “empty” plots with perennial or non-tree crops. The two farms differed in their spatial arrangement, size and age of the host and non-host trees (Figure 1).

For both farms, the 15th of May was set as the first day for all simulations. However, the time of the fly emergence and the onset of the cherry season varied between the two locations by ca. 14 days, according to their latitudinal difference, and alike, the phenological type and composition of the main cherry cultivars. Thus, for the BOKU farm, Burlat, Blaze Star, Kordia, and Regina were used to represent the main four phenological groups. Burlat was harvested on 4th June, Blaze Star on 18th June, Kordia partially on 25th June and later on 2nd July, Regina on 2nd and 9th July. On the PC-Fruit farm, only medium and late fruiting cultivars were grown, the latter included also some “old” unidentified sweet cherry cultivars. Accordingly, Kordia, Lapins, Regina, and Sweetheart were used as the main phenological representatives. Kordia was harvested on 9th July, Lapins on 18th July, Regina on 25th July, and Sweetheart on 29th July.

On both farms, natural, and well-established *R. cerasi* populations were present, but their overall densities and temporal patterns varied substantially. With the same number (8) of the Rebel traps set on each farm, during the season, 953 and 123 females were caught, and the maximum catch (on control plots) was recorded on 11th June and on 16th July, on BOKU and PC-Fruit farm, respectively. On both farms, *R. cerasi* was controlled on one plot only, and no other IPM treatments against the pest were made on the farm remainder. In BOKU, a pesticide was applied only once, on the plot (ca. 0.3 ha) with trap No 7, containing (almost exclusively) Kordia and Regina cultivars. The pesticide, Mospilan 20 SG (0.0375%), was cover-sprayed on 5th June, at the time of the fruit color change on Kordia (from green to yellowish-green). The same cultivars, Kordia and Regina, were present on the protected plot (ca. 0.35 ha) on the PC-Fruit farm. Part of the plot, containing traps No 6 & 7, was spot-sprayed with a mixture of a bait and pyrethroid, and the part containing the trap No 8 was cover-sprayed with Spinosad. The treatments were repeated twice, on 16th and 30th of June.

**Figure 3:**
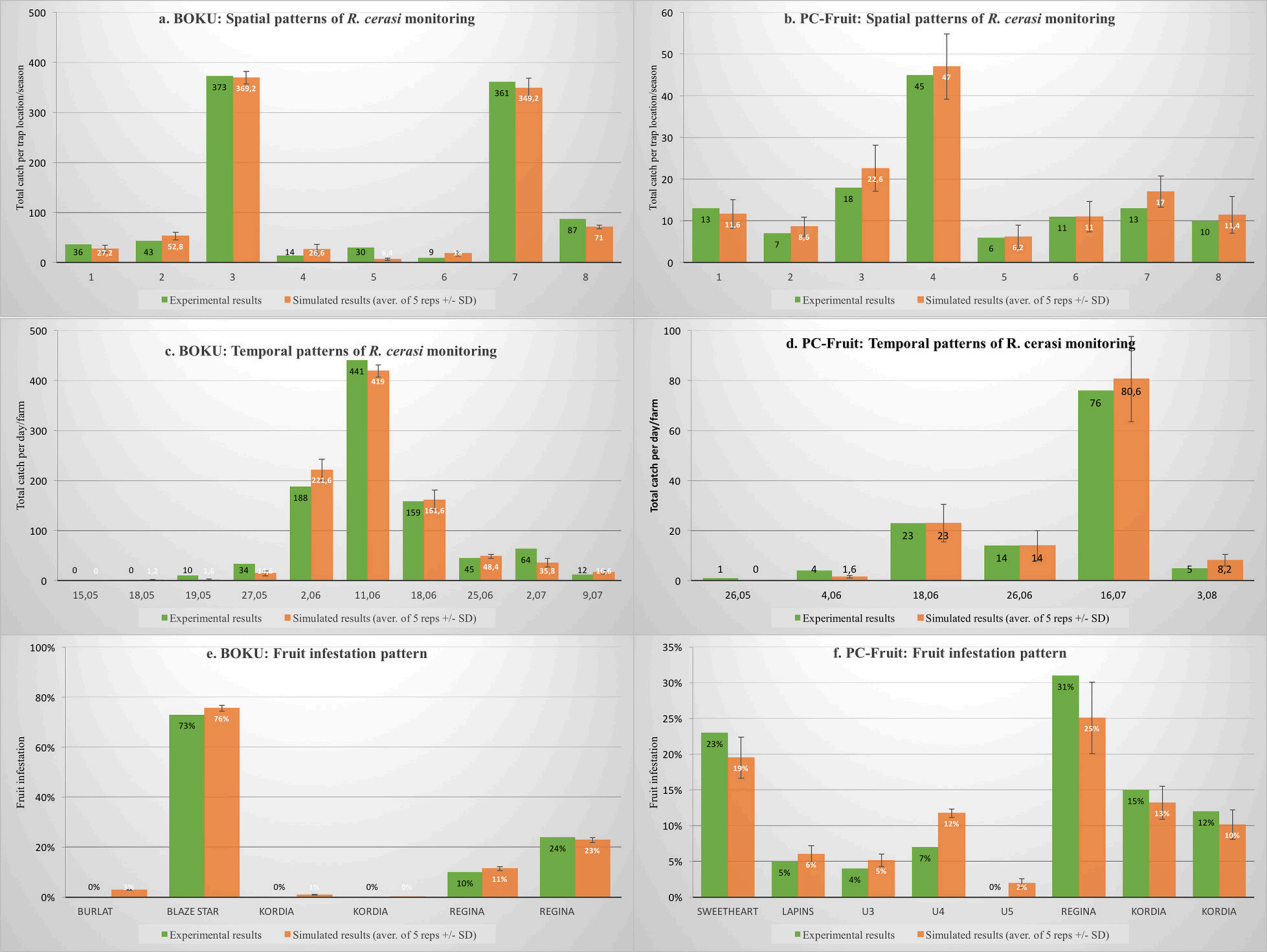
**On-farm IPM experiments: comparison between experimental vs. model-generated spatial and temporal pest monitoring and fruit infestation patterns**.

Spatial and temporal patterns of the trap catches during the pest monitoring conducted on each farm and the model-generated simulation results are presented on Figures 3A–D. In spite of substantial differences between the two farms, the simulated pest monitoring largely conformed to that obtained on-farm. No significant discrepancy between the overall patterns was detected (Table 4), and most of the data points passed the simplified process control test and fell within the respective 3-sigma control limits. However, failure to detect discrepancy in the overall patterns has to be interpreted cautiously, especially when some trap catches are inherently low and erratic, such as early and late in season, or in the traps located outside of the host zones. Indeed, in some of such cases, experimental results fell outside of the 3-sigma control limits (Figure 3A Trap 5; Figure 3C catches on: 19.05 & 2.07; Figure 3D catches on 4.06).

**Table 4:** **Comparison between pest monitoring and fruit infestation patterns: experimental vs. model-generated**.

**Source data**	**Chi-square**	**df**	***p*-value**
Figure 3A. BOKU: spatial pattern of pest monitoring	1.364	7	0.2582
Figure 3C. BOKU: temporal pattern of pest monitoring	2.541	9	0.1124
Figure 3B. PC-Fruit: spatial pattern of pest monitoring	3.011	7	0.4721
Figure 3D. PC-Fruit: temporal pattern of pest monitoring	0.875	5	0.8541
Figure 3E. BOKU: fruit infestation pattern	1.718	5	0.4217
Figure 3F. PC-Fruit: fruit infestation pattern	2.321	7	0.3547

Comparison of the experimental and simulated fruit infestation patterns, for each of the four representative cultivars at their respective harvest times, is presented on Figures 3E,F. Simulation of fruit infestation is prone to errors. While numbers of eggs laid in each plot or farm sector are emulated with fair accuracy, their translation into the fruit infestation depends on the precision of the local fruit load (productivity) estimation. Experimental assessment of the actual fruit productivity and infestation is laborious, therefore the calculation was based on quite broad estimations, generated by the model, and compared with a limited number of experimental records. Nevertheless, both for the control and the pesticide-treated plots, and regardless of cultivar phenology and harvest time, the simulated results of fruit infestation conformed acceptably to the experimental data. No significant discrepancy between the overall patterns was detected (Table 4), and only a few data points, all with low and thus erratic infestation, fell outside the respective 3-sigma control limits (Figure 3E Burlat 4th June and Kordia 25th June; Figure 3F U4).

Admittedly, the appearance of some data points outside the 3-sigma control limits indicates likely imperfections of the simulation process. On the other hand, it is worth to emphasize that the detected discrepancies were numerically small, related to the inherently erratic data, thus were of very limited practical importance. The overall similarity of the simulated and experimental results corroborates, that the model emulated the key on-farm processes, such as phenology of various host cultivars, patterns of pest emergence and mortalities, its on-farm movements and fruit infestation, and also IPM actions—pest monitoring (for specific trap locations) and effects of the local pesticide application. The results indicate that the model-based “virtual farms” constitute acceptable representation of the respective real farms, where the IPM experiments were conducted.

## Discussion

Agent-based models permit incorporation of a large number of component processes, which determine behavior of their agents, or just modify it under certain circumstances. Consequently, model adaptation constitutes critical, but also an open-ended process, mirroring the current status and progress in our understanding of the causative agent (here: *R. cerasi*). The choice of the processes to be modeled, and the quality of the input information—jointly determine the relevance and precision of the model. Explicably, in simulation of complex systems, rigorous evaluation of all assumed relations and parameters may not be feasible. The purist approach—incorporating into the model only rigorously established and parametrized processes, although tempting and warranting formal methodological correctness, also comes at a price. Discounting plausible, but superficially quantified aspects of biology *de facto* entails adopting “hidden,” and frequently much less correct, default patterns for the “discarded” processes—a zero-order linear relations.

Being faced with such dilemma, we have chosen a pragmatic approach—including into the model also putative, and in some cases, only tentatively parametrized processes, in order to construct a “frame-model” capable to provide initially-acceptable emulation of the system. Indeed, the experience shows, that viable agent-based models, generating plausible answers to complex questions, can be constructed even in the shortage of detailed knowledge about the system (An et al., 2009). Later on, with new information, the rules and parameters can be fine-tuned, without having to modify the entire model.

Accordingly, a simple approach to model validation was taken—testing whether the model can reproduce empirical data with reasonable accuracy. The results, presented above, confirmed the general capacity of the model to emulate the key on-farm processes and satisfactorily reproduce IPM experiments conducted on the respective farms. But ultimately, the model value rests in its ability to extrapolate to situations beyond those originally observed on-farm, to provide new insights extending beyond what was already known. Results of such applications of the model are discussed below.

### Insights into the seasonal patterns of pest population density

**Figure 4:**
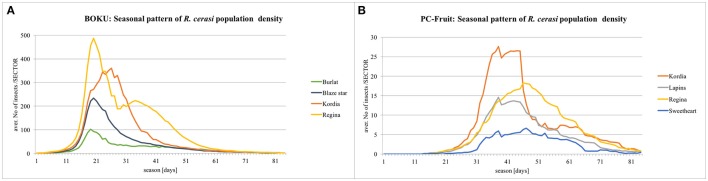
**Simulated seasonal patterns of ***R. cerasi*** population density**. Assumption: NO pesticide treatment on the whole farm.

Seasonal patterns of *R. cerasi* population density, simulated under assumption of NO pesticide application on the whole farm, are presented on Figure 4. In BOKU, the simulated peak of population density occurred during 20th–22nd season day (3rd–5th June), although a sizeable population continued until 50th day (on late cultivars). In PC-Fruit, the population culminated around 35th day and, due to more extended and “flat” profile of pest emergence from the soil in spring, continued at such high level until ca. 45th day, afterwards gradually declined, but still a sizeable population continued beyond 75th day.

The difference between the farms in the simulated and on-farm recorded profiles of fly emergence was probably caused by more diversified topography and ground cover of the PC-Fruit farm, and generally lower temperatures prevailing during the pest emergence—from 5th till 40th season day (19th May–23rd June) the average daily temperature was 3°C lower (22.1 and 19.1°C, BOKU and PC-Fruit, respectively). Furthermore, in PC-Fruit only medium and late maturing cultivars are grown, characterized by occurrence of late and prolonged “fruit suitability windows” (Table 2), hence a degree of the local pest adaptation (delayed and diffused emergence to cover delayed and longer fruit suitability)—appears likely.

In general, the European cherry fruit fly is a “sedentary” pest, closely linked to its host tree (especially when isolated), with relatively limited propensity for distant translocations (Daniel and Grunder, 2012). However, within the plots with continuous canopy coverage, local explorations and transfers “from tree to tree” are common (Wiesmann, 1935; Łȩski, 1963; Daniel and Wyss, 2009). Thus, when two or more cultivars, substantially different in their phenology and the extent of “fruit suitability windows,” are present on the same plot, local shifts “within-the-plot” are likely. Indeed, on the simulated patterns, both for BOKU and PC-Fruit farm, on the plots containing Kordia and Regina cultivars, a degree of a local shift of the pest is visible (Figure 4), with the majority seen first on Regina, shifting later toward Kordia at the peak of its fruit suitability. When Kordia fully ripened and the fruit became dark (nearly black) and thus less attractive, the flies shifted back to the later ripening Regina, having still suitable and attractively red fruit.

Transfers among the plots within the farm, although occur and become progressively more frequent with advancing female age and decreasing availability of suitable fruit, are generally limited. In consequence, substantial differences can be maintained in the density of the resident pest populations among the isolated plots containing various cherry cultivars. The local density is relative to the capacity of the cultivar to sustain complete in-fruit immature development cycle, which is much higher for later maturing cultivars with more extended “fruit suitability window,” better correlated with the period of the pest's peak fecundity.

All these phenomena are well reflected by the simulated results, but importantly, the model allows to assess their local magnitude and implications for various IPM scenarios.

### Insights into the impact of cultivar phenology on the effective female fecundity

The potential lifetime fecundity (365 eggs/female) adopted for model calibration, based on the laboratory data obtained under optimal conditions, unlimited food and fruit supply and the absence of any extrinsic mortality causes (Köppler et al., 2008; Moraiti et al., 2012), may appear inconsistent with the reports about the on-farm estimated effective fecundity (30–200 eggs/female; Łȩski, 1963; Daniel and Grunder, 2012). However, alike on a real farm, the “virtual” females are also exposed to a daily changing configurations of various mortality risks, are challenged by the necessity to survive 5–10 days to reach maturity and attain the capacity for egg-laying, are also subject to individual aging and resultant changes in fecundity, and often, faced with imperfect alignment between the periods of their peak fecundity and the local fruit “suitability windows.” Thus, the simulated effective average fecundity of a “virtual” female newly emerging from the soil in spring was strongly dependant on the phenology of the cultivars prevailing on the plot—and ranged from less than 1 on Burlat, ca. 6 on early cultivars up to ca. 27 eggs/female on the late ones. As expected, IPM treatments reduced lifespan on the females dwelling on the treated plots, and accordingly, their net fecundity, but the model allowed for approximate quantification of such effects according to the local conditions (Table 5).

**Table 5:** **Influence of host phenology and IPM treatments on the effective net fecundity of ***R. cerasi*** females**.

Net effective fecundity/female emerging from the soil in spring^*^									
**BOKU**					**PC-Fruit**				
**Sweet cherry cultivar**	**NO pesticide^A^**		Pesticide treatment^B^^**^		**Sweet cherry cultivar**	**NO pesticide^A^**		Pesticide treatment^A^^**^	
	**Aver**.	**SD**	**Aver**.	**SD**		**Aver**.	**SD**	**Aver**.	**SD**
Burlat	0.69^c^	0.06	0.56^c^	0.02	Lapins	12.25^c^	0.65	11.90^b^	0.18
Blaze star	6.19^b^	0.05	5.51^b^	0.07	Sweetheart	25.50^a^	2.29	25.39^a^	1.19
Kordi^a^	24.90^a^	0.16	**8.60**^a^	0.08	Kordi^a^	7.68^d^	0.15	**5.28**^c^	0.39
Regin^a^	26.89^a^	0.15	**9.84**^a^	0.18	Regin^a^	20.90^b^	0.38	**14.47**^b^	0.20

* *Different capital letters indicate significant differences between respective NO pesticide and Pesticide treatment; different small letters indicate significant differences between cultivars*.

** *Pesticide treatments are indicated by bold letters*.

### Insights into seasonal modulation of pest age structure

**Figure 5:**
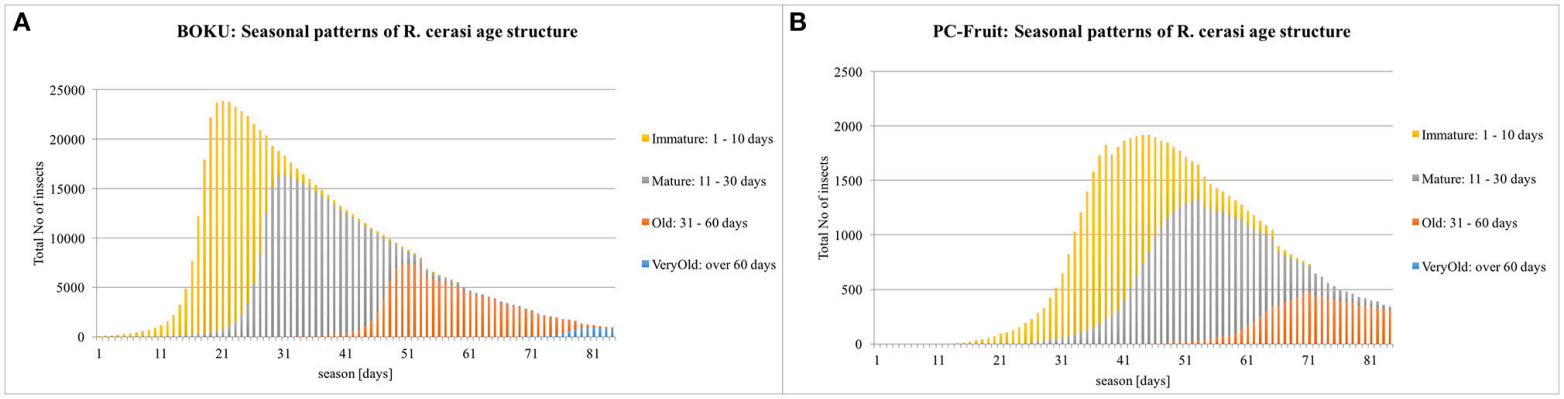
**Simulated seasonal changes in ***R. cerasi*** age structure**. Assumption: NO pesticide treatment on the whole farm.

The Figure 5 shows the timing and cumulative density (for the whole farm) of the main four female age categories; immature (1–10 day-old), mature (11–30 days-old), old (31–60 days-old) and senile (over 60 days-old), simulated under the assumption of NO pesticide application on the whole farm. The overall seasonal patterns of female age structure differ between the two farms. In BOKU, fairly clear succession of the age cohorts can be seen, in contrast to extensive overlap and largely concurrent presence in PC-Fruit.

The four female age categories differ in their behavior, fecundity, and thus importance from the “farmer's point of view.” Immature females, although present on farm and trapped during monitoring, are unable to infest the fruit and thus do not pose any threat to the crop. At this stage, their exploratory mobility is reduced. Maturing females increase their propensity for local explorations, and soon after, attain the peak of their reproductive capacity. This stage is the most damaging and thus should become the primary target of any IPM. Old females still have substantial fecundity and thus crop damaging potential, and enhanced propensity for longer explorative errands. However, after over 30 days of exposure to numerous mortality risks, their population is already decimated, and therefore, of lesser practical importance. The oldest category, over 60-days-old, although retaining some “residual” fertility, due to very low densities and probably reduced responsiveness to Rebel traps, are barely visible on farm and their practical impact is negligible.

The simulation indicates, the main IPM effort shall be focused from ca. 20th till 45th season day in BOKU, and for much longer period, from ca. 30th till 75th season day, in PC-Fruit.

### Insights into mechanisms of the local IPM

The IPM regime applied in BOKU—a single cover-spray with a systemic pesticide, Mospilan (acetamiprid)—targeted primarily the immature stages developing in the fruit, though the pesticide has also short-term knockdown action against the adults. The pesticide was applied at the time of fruit-color-change in Kordia. Although the treatment ensured weeks-long systemic action, effectively killing the immature stages developing in the fruit, its efficacy gradually decreased before the harvest. Nevertheless, the protection was effective for Kordia, but the residual pesticide activity was insufficient to prevent substantial infestation of the later ripening Regina. Simulations of the on-farm experiment reveal, that if the daily larval mortality rate in the fruit (caused by the systemic pesticide) drops below ca. 60% during a few days (4–8) before the harvest, substantial crop damage is unavoidable. When a complete removal of this residual protection was simulated for the last 7 days, the infestation of Regina jumped to ca. 68%, compared to 24% recorded on the farm. The results explain why, with the IPM relying on the use of systemic pesticides and targeting the immature stages of the pest, it is virtually impossible to produce a “maggot-free” and truly “pesticide-free” fruit at the same time. This difficulty was recognized by the growers, who prompted EFSA to re-evaluate the formal EC MRL (Maximum Residue Level) for acetamiprid residues in sweet cherries, and increase the threshold from the earlier 0.2 mg/kg to 0.5 mg/kg (EFSA, 2010).

**Figure 6:**
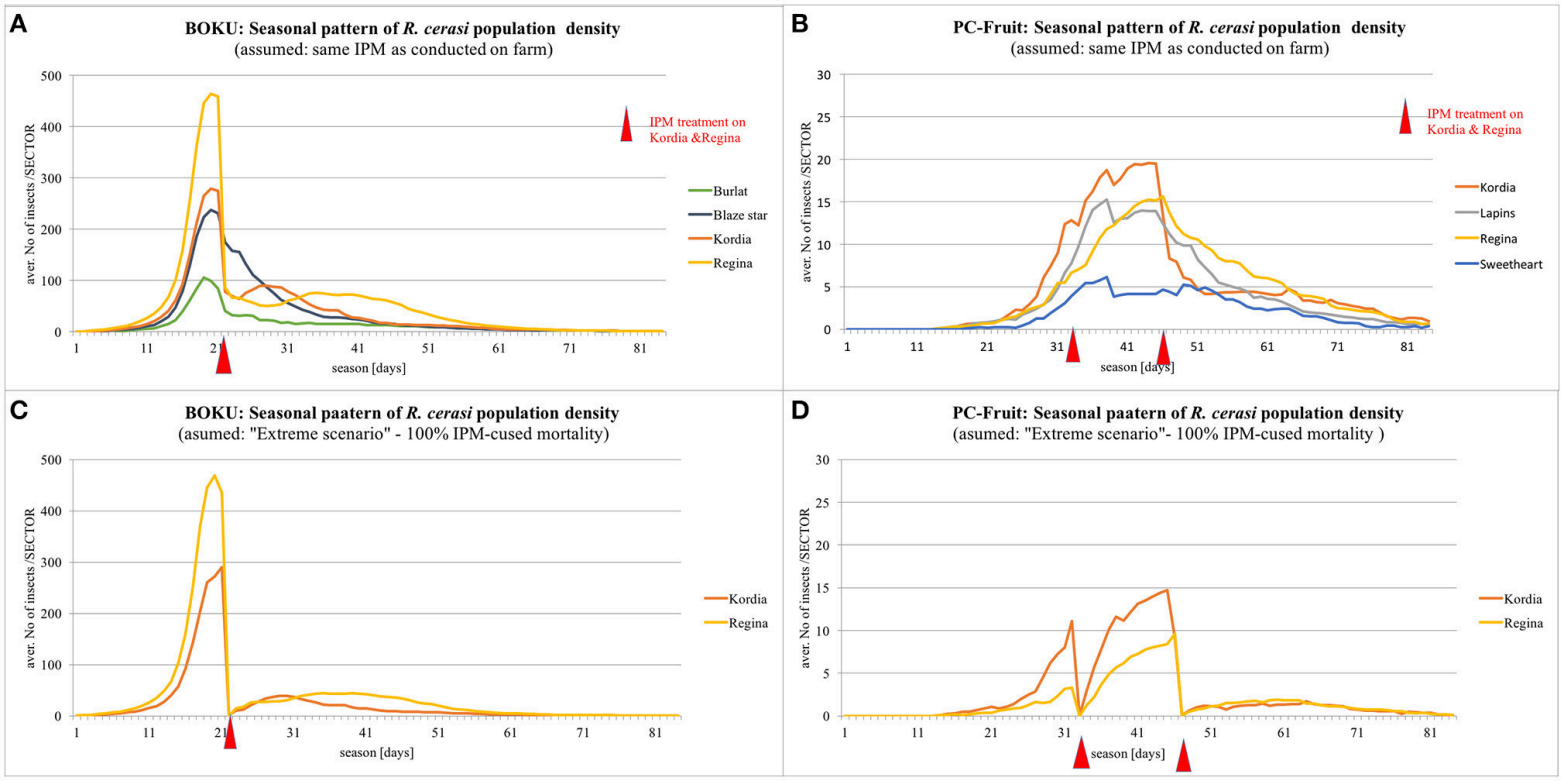
**Reduction and resurgence of ***R. cerasi*** population after IPM treatments**.

On both farms, the adult populations were substantially reduced by IPM programmes (single spray in BOKU and 2 treatments in PC-Fruit, both on the plots containing Kordia and Regina cultivars; Figures 6A,B) compared to the “NO-treatment” scenario (Figures 4A,B). However, they failed to eliminate the flies entirely, and prevent crop infestation. Even when a more extreme scenario was simulated—assuming complete absence of the pest on all other cherry plots except the ones treated with a pesticide, and on the latter, application of a short-acting (1 day only), knockdown, non-systemic pesticide inflicting 100% mortality to the adults present on the plot, applied in the same regime (timing and repetitions) as the experimental IPM treatment—still post-treatment pest populations re-appeared on the treated plots (Figures 6C,D) and caused crop damage (ca. 40–50% of that recorded the on-farm), both in the case of BOKU and PC-Fruit.

The source of the post-treatment population is not immediately clear, it may originate either from active immigration of females from the nearby plots, and/or “from the local soil”—through late emergence of the flies still present on the plot. To assess the relative contribution of the two “resurgence” pathways, several scenarios were simulated for each of the two farms, assuming various combinations of the presence or absence of flies on the treated and/or on all other (NON-treated) plots. The IPM treatment, if deemed applied, was always the same as the experimental treatments applied during the field experiments on the respective farms. The results, presented in Table 6, reveal that both in BOKU and PC-Fruit case, the major source of the post-treatment population increase was present on the treated plot—the late flies emerging from the soil after the treatment. The impact of this phenomenon on the effectiveness of IPM was more acute on the PC-Fruit farm, due to delayed and more prolonged fly emergence process. Relative contribution of the flies immigrating from the nearby plots, although considerable, was of lesser importance, and was dependent on the local farm configuration and distances to the nearby plots.

**Table 6:** **Contribution of various pest sources to the post-treatment population resurgence**.

**Assumed scenario**				**Fruit infestation (Regina cultivar)**			
**Scenario ID**	**Pest source**		**IPM treatment**	**BOKU**		**PC-Fruit**	
	**The treated plot**	**NON-treated plots**		**Aver. (%)**	***SD*** **(%)**	**Aver. (%)**	***SD***
a	x	x	x	22.84	0.87	25.08	4.49%
b	–	x	X	5.91	0.88	3.37	0.99%
c	X	–	X	17.87	0.84	28.24	3.55%
d	x	x	–	43.01	1.68	49.30	7.02%

This phenomenon, frequently neglected or not fully realized, explains the challenges faced by the IPM programmes targeting only the adult flies, and reveals the importance of taking into account the local farm specificity in designing IPM strategy.

### Insights into the carry-over of pest population–the next season outlook

Understanding the local carry-over mechanism of the pest population to the next season is of utmost importance for successful IPM. The European cherry fruit fly, being a univoltine insect, has no capacity to multiply its adult population during the season, thus the carry-over of the immature stages pupating in the soil entirely determines the level of the next season threat the pest can pose to the crop. Although, when mature, the larvae actively depart from the fruit and jump into the soil, it is widely believed that harvest is one of the primary mechanisms for mass-removal of larvae from the orchard and thus one of the main mortality factors (Boller, 1966; Daniel and Grunder, 2012). Consequently, complete harvest is recommended as a measure of pest management. However, the extra effort and cost to increase the harvest accuracy from the usual ca. 80% to the required 100% is prohibitively high, which is the reason for low adoption of this measure by fruit growers.

**Figure 7:**
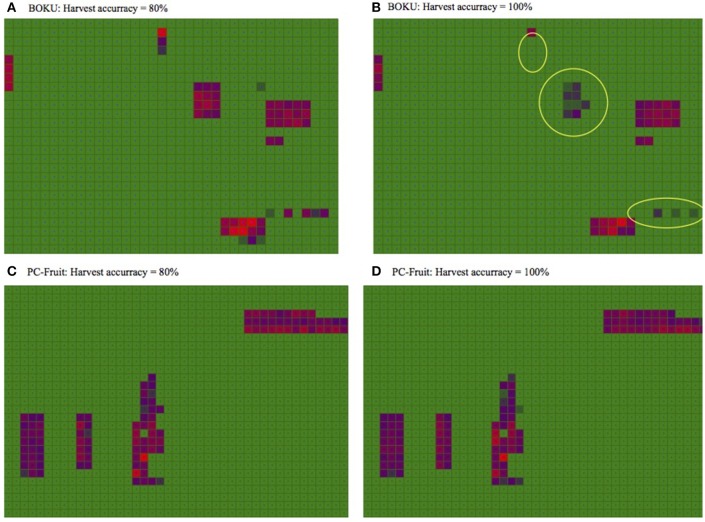
**Impact of harvest accuracy on the carry-over of pest population and anticipated emergence next spring**. On the diagrams, intensity of the red hue corresponds to the local density of overwintering pupae.

To visualize the impact of harvest completeness on the carry-over of fly population in relation to phenology of the on-farm present sweet cherry cultivars, two scenarios were simulated for each of the farms: assuming either 80 or 100% harvest accuracy, with the harvest conducted at the same time and with the same IPM regime as that applied during the on-farm experiments. The results, presented on Figure 7, show that the impact of the complete harvest is substantial and clearly visible (yellow frames) only in the case of the medium-maturing cultivars, present in BOKU. But even on this farm, the difference for late varieties is negligible. On PC-Fruit, where late cultivars were grown, most of the larvae completed development and left the fruit before harvest, hence its completeness appears to have no practical bearing on the next year population.

Notwithstanding the potential of this measure when applied on early and medium maturing cultivars, in the light of our results, the universal value of the recommended harvest completeness appears questionable, especially in the areas where late cultivars are predominantly grown.

## Concluding remarks

The model represents bottom-up “ethological” approach to the site-specific IPM, focused on behavior of the individual insects—the primary actors determining local IPM performance. The results demonstrate that large amounts of quantified information about various aspects of the local farming system, pest biology and behavior can be consolidated and embedded into the model, and converted into an operable site-specific IPM enhancement tool. Although, still imperfect, the model generates projections closely mirroring the results of the on-farm conducted IPM experiments. The model has in-built provisions to absorb new and more detailed pest-relevant and farm-specific information, and thus gradually improve its local relevance and robustness of its projections.

In its current state, it constitutes a viable “virtual” representation of the target sites and allows for “virtual” evaluation of numerous site-specific IPM scenarios. Admittedly, implementation of such tool will not eliminate field experiments, but can radically shorten the usual “development trajectory” by substituting major part of long-term and expensive on-farm experiments by their “virtual” emulations.

The “virtual insect” module was designed in a generic from, and can be adapted to a number of insect species of various biology. Its variants adapted to the target insect (pest) become strictly species specific, and can be used to drive any number of “virtual farm” sub-modules, where the same pest is the causative agent. The process of adaptation, both to the insect and to the specific farm, is largely “researcher driven.” But once the farm is characterized and the model tuned to the local conditions, unlimited number of site-specific pest management scenarios can be modeled and evaluated. At this stage, the farmer can take the lead in formulation of various IPM scenarios, and make management decisions based on the received assessment of their efficacy and cost/benefit. To compare the effects of the chosen scenario with the results of its implementation on-farm, the farmer will have to provide information very similar to that normally collected, such as pest monitoring data, records about the IPM treatments, host tree phenology, fruit infestation and yield.

For each scenario, the simulation process generates substantial volume of information, normally not accessible during experimental work, which provides insights into the processes operating on the particular farm, such as seasonal patterns of pest emergence, density and age structure, seasonal patterns of pest mortality caused by aging, natural enemies, bad weather spells, pesticides, trapping etc., phenology for the main host cultivars, spatial patterns of insect translocations within the farm, emerging patterns of fruit infestation, anticipated patterns of female emergence next season, etc. A typical output, generated by the model after each simulation run, is presented in the Complementary materials.

A converse application of the model is also possible, for modification of the farm topography and development of pest-resilient landscape and site-specific IPM. The model also has the potential for conversion into a site-specific forecasting tool, once more detailed and complete information about the impact of climate on the host phenology and pest behavior becomes available.

## Author contributions

SL conceived the project, contributed proprietary generic PESTonFARM model, made all model adaptations (conceptual modifications, algorithm and code writing, assumptions and calibrations), executed and interpreted all the simulations used in the manuscript. AW conducted on-farm surveys and detailed local inventories for all sites, and executed the on-farm observations and experiments on *R. cerasi* behavior and ecology. HV provided access to extensive data, including historic information about experiments conducted in the past at JKI on *R. cerasi* ecology, and supplied insect material (*R. cerasi* pupae) for all the experiments. TB, AS provided access to historic data about *R. cerasi* IPM and ecology relative to the PC-Fruit and BOKU sites, respectively, and conducted the local on-farm IPM experiments and observations on sweet cherry phenology. MS provided methodological comments and executed all statistical analyzes in discussion with the remaining authors. SL wrote the first draft of the manuscript, and all authors contributed to the final version.

### Conflict of interest statement

The authors declare that the research was conducted in the absence of any commercial or financial relationships that could be construed as a potential conflict of interest.

## Footnotes

^2^Directive 2009/128/EC of the European Parliament and of the Council of 21 October 2009. Official Journal of the European Union L309/71, 24.11.2009 http://www.eppo.int/PPPRODUCTS/information/2009_0128_EU-e.pdf

## References

[B1] An G. Mi Q. Dutta-Moscato J. Vodovotz Y. (2009). Agent-based models in translational systems biology. Syst. Biol. Med. 1, 159–171. 10.1002/wsbm.45 PMC3640333 20835989

[B2] Böckmann E. Hummel E. Vogt H. (2012). Promising field and semi field results for cherry fruit fly control using neem, in 15th International Conference on Organic Fruit-Growing: Proceedings to the Conference from February, 20 to February, 22, 2012 at Hohenheim Germany (Weinsberg: FOEKO), 167–172.

[B3] Böckmann E. Köppler K. Hummel E. Vogt H. (2014). Bait spray for control of European cherry fruit fly – an appraisal based on semi-field and field studies. Pest Manag. Sci. 70, 502–509. 10.1002/ps.3621 23893955

[B4] Boller E. (1966). Beitrag zur Kenntnis der Eiablage und Fertilität der Kirschenfliege *Rhagoletis cerasi* L. Mitt. Schweiz. Entomol. Ges. 38, 195–202.

[B5] Boller E. (1969). Neues über die Kirschenfliege: freilandversuche im Jahr 1969. Schweiz. Z. Obst-und Weinbau 105, 566–572.

[B6] Boller E. F. (1966). Der Einfluss natürlicher Reduktionsfaktoren auf die Kirschenfliege *Rhagoletis cerasi* L. in der Nordwestschweiz, unter besonderer Berücksichtigung des Puppenstadiums, Doctoral dissertation, Diss. Techn. Wiss. ETH Zürich.

[B7] Daniel C. Baker B. (2013). Dispersal of *Rhagoletis cerasi* in commercial cherry orchards: efficacy of soil covering nets for cherry fruit fly control. Insects 4, 168–176. 10.3390/insects4010168 26466801 PMC4553435

[B8] Daniel C. Grunder J. (2012). Integrated management of European Cherry fruit fly *Rhagoletis cerasi* (L.): situation in Switzerland and Europe. Insects 3, 956–988. 10.3390/insects3040956 26466721 PMC4553558

[B9] Daniel C. Wyss E. (2009). Migration und ausbreitung der kirschfruchtfliege innerhalb von obstanlagen-möglichkeit der biologischen bodenbehandlung, in Proceedings of the 10th Wissenschaftstagung Ökologischer Landbau, eds Mayer J. Alföldi T. Leiber F. Dubois D. Fried P. Heckendorn F. Hillmann E. Klocke P. Lüscher A. Riedel S. Stolze M. Strasser F. van der Heijden M. Willer H. (Berlin: Verlag Dr. Köster), 300–301.

[B10] DeAngelis D. L. Grimm V. (2014). Individual-based models in ecology after four decades. F1000Prime Rep. 6:39. 10.12703/P6-39 24991416 PMC4047944

[B11] DeAngelis D. L. Mooij W. M. (2005). Individual-based modelling of ecological and evolutionary processes. Annu. Rev. Ecol. Evol. Syst. 36, 147–168. 10.1146/annurev.ecolsys.36.102003.152644

[B12] EFSA (2010). Reasoned opinion of EFSA. modification of the existing MRL for acetamiprid in cherries. EFSA J. 8:1494 10.2903/j.efsa.2010.1494 Available online at: http://www.efsa.europa.eu/en/efsajournal/pub/1494

[B13] European Commission DG SANCO. (2013). Ad-hoc Study to Support the Initial Establishment of the List of Candidates for Substitution as Required in Article 80(7) of Regulation (EC) No 1107/2009. Final report. Brussels: The Food Chain Evaluation Consortium (FCEC), Civic Consulting, Agra CEAS Consulting, Van Dijk Management Consultants, Arcadia International. Available online at: http://ec.europa.eu/food/plant/pesticides/approval_active_substances/docs/cfs_final_report_072013_en.pdf

[B14] Fajardo A. (2009). Beyond description: the active and effective way to infer processes from spatial patterns. Ecology 90, 46–56. 10.1890/07-2096.1 19294912

[B15] Grimm V. Augusiak J. Focks A. Frank B. M. Gabsi F. Johnston A. S. A. (2014). Towards better modelling and decision support: documenting model development, testing, and analysis using TRACE. Ecol. Modell. 280 129–139. 10.1016/j.ecolmodel.2014.01.018

[B16] Grimm V. Berger U. Bastiansen F. Eliassen S. Ginot V. Giske J. (2006). A standard protocol for describing individual-based and agent-based models. Ecol. Modell. 198, 115–126. 10.1016/j.ecolmodel.2006.04.023

[B17] Grimm V. Berger U. DeAngelis D. L. Polhill J. G. Giske J. Railsback S. F. (2010). The ODD protocol: a review and first update. Ecol. Modell. 221, 2760–2768. 10.1016/j.ecolmodel.2010.08.019

[B18] Grimm V. Railsback S. F. (2005). Individual-based Modeling and Ecology. Princeton, NJ: Princeton University Press.

[B19] Grimm V. Revilla E. Berger U. Jeltsch F. Mooij W. M. Railsback S.. (2005). Pattern-oriented modelling of agent-based complex systems: lessons from ecology. Science 310, 987–991 10.1126/science.1116681 16284171

[B20] Jovani R. Grimm V. (2008). Breeding synchrony in colonial birds:from local stress to global harmony. Proc. R. Soc. B 275, 1557–1563. 10.1098/rspb.2008.0125 18397868 PMC2602658

[B21] Katsoyannos B. I. (1975). Oviposition-deterring, male-arresting, fruit-marking pheromone in *Rhagoletis cerasi*. Environ. Entomol. 4, 801–807. 10.1093/ee/4.5.801

[B22] Katsoyannos B. I. (1979). Zum Reproduktions-und Wirtswahlverhalten der Kirschenfliege, Rhagoletis cerasi L.(Diptera: Tephritidae). Doctoral dissertation, Diss. Techn. Wiss. ETH Zürich.

[B23] Katsoyannos B. I. Boller E. F. Benz G. (1986). Verhalten der Kirschenfliege, Rhagoletis cerasi L., bei der Auswahl der Wirtspflanzen und ihre Dispersion. Mitt. Schweiz. Entomol. Ges. 59, 315–335.

[B24] Köppler K. Féjoz B. Vogt H. (2010). Correlation between maturity of female *R. cerasi*, oviposition, larval development and ripeness of cherries. Int. Fruit Prot. Fruit Crops IOBC/wprs Bull. 54, 663–667.

[B25] Köppler K. K. Kaffer T. Vogt H. (2008). Bait sprays against the European cherry fruit fly *Rhagoletis cerasi*: Status Quo & Perspectives, in Ecofruit - 13th International Conference on Cultivation Technique and Phytopathological Problems in Organic Fruit-Growing: Proceedings to the Conference from 18thFebruary to 20th February 2008, ed Boos M. (Weinsberg), 102–108.

[B26] Lazić S. Šunjka D. Paniæ S. Inğiæ D. Grahovac N. Guzsvány V. (2014). Dissipation rate of acetamiprid in sweet cherries. Pestic. Phytomed. 29, 75–82. 10.2298/PIF1401075L

[B27] Łȩski R. (1963). Studia nad biologia i ecologia nasionnicy tzresniowki *Rhagoletis cerasi* L. (Diptera: Trypetidae). Pol. Pismo Entomol. Ser. B 3, 153–240.

[B28] Lux S. A. (1989). Stochastic model of the Khapra beetle, Trogoderma granarium Everts reproductive behaviour, in International Ethological Conference XXI, 9-17 August (Utrecht).

[B29] Lux S. A. (1992). Diagnosis of behaviour as a tool for quality control of mass reared arthropods, in Proceedings of the Fifth Workshop of the IOBC Global Working Group ‘Quality Control of Mass Reared Arthropods’: Wageningen, ed Bigler F. (Zurich: Swiss Federal Research Station for Agronomy), 66–79.

[B30] Lux S. A. (1994). Ethological aspects of rearing insects, in Techniques of Insect Rearing for the Development of Integrated Pest and Vector Management Strategies. Vol. 1, ed Ochieng-Odero J. P. R. (Nairobi: ICIPE Science Press), 173–186.

[B31] Lux S. A. (2014). PESTonFARM - stochastic model of on-farm insect behaviour and their response to IPM interventions. J Appl. Entomol. 138, 458–467. 10.1111/jen.12083

[B32] Lux S. A. Gaggl K. (1996). Ethological analysis of medfly courtship: potential for quality control, in Fruit Fly Pests: A World Assessment of Their Biology and Management, eds McPheron B. A. Steck G. J. (Delray Beach, FL: St. Lucia Press), 425–432.

[B33] McAtee P. Karim S. Schaffer R. David K. (2013). A dynamic interplay between phytohormones is required for fruit development, maturation, and ripening. Front. Plant Sci. 4:79. 10.3389/fpls.2013.00079 23616786 PMC3628358

[B34] Moraiti C. A. Nakas C. T. Papadopoulos N. T. (2012). Prolonged pupal dormancy is associated with significant fitness cost for adults of *Rhagoletis cerasi* (Diptera: Tephritidae). J. Insect Physiol. 58, 1128–1135. 10.1016/j.jinsphys.2012.05.012 22684113

[B35] Ozdem A. Kilincer N. (2008). The biology of the European Cherry Fruit fly [*Rhagoletis cerasi* L. (Diptera: Tephritidae)]. Acta Hort. 795, 897–904. 10.17660/ActaHortic.2008.795.145

[B36] Parker D. C. (2005). Agent-based modelling to explore linkages between preferences for open space, fragmentation at the urban-rural fringe, and economic welfare, in Paper Presented at The Role of Open Space and Green Amenities in the Residential Move from Cities, December 14–16 2005, Dijon.

[B37] Parker D. C. Berger T. Manson S. M. (2002). Agent-Based Models of Land-Use and Land Cover Change: Report and Review of an International Workshop, October 4-7, 2001. LUCC Report Series No. 6. Anthropological Center for Training and Research on Global Environment Change, Indiana University, LUCC Focus 1 Office, Irvine, CA.

[B38] Polhill J. G. Parker D. C. Brown D. G. Grimm V. (2008). Using the ODD protocol for describing three agent-based social simulation models of land-use change. J. Artif. Soc. Soc. Simul. 11, 3 Available online at: http://jasss.soc.surrey.ac.uk/11/2/3.html

[B39] Prokopy R. J. (1968). Orientation of the apple maggot flies *Rhagoletis pomonella* (Walsh) and European cherry fruit flies *R. cerasi* L. (Diptera: Tephritidae) to visual stimuli, in Proceedings of the 13 International Congress of Entomology (Moscow), 34–35.

[B40] Prokopy R. J. Papaj D. R. Opp S. B. Wong T. T. Y. (1987). Intra-tree foraging behavior of *Ceratitis capitata* flies in relation to host fruit density and quality. Entomol. Exp. Appl. 45, 251–258.

[B41] Reed M. Alvarez T. Chelinho S. Forbes V. Johnston A. Meli M.. (2016). A risk assessment example for soil invertebrates using spatially explicit agent-based models. Integr. Environ. Assess. Manag. 12, 58–66. 10.1002/ieam.1713 26411378

[B42] R Development Core Team (2015). R: A Language and Environment for Statistical Computing. Available online at: http://www.r-project.org/

[B43] Schumann C. Schlegel H. J. Grimm E. Knoche M. Lang A. (2014). Water Potential and its components in developing sweet cherry. J. Amer. Soc. Hort. Sci. 139, 349–355.

[B44] Senger S. E. Tyson R. C. Roitberg B. D. Thistlewood H. M. A. Harestad A. S. Chandler M. T. (2009). Influence of habitat structure and resource availability on the movements of *Rhagoletis indifferens*. Environ. Entomol. 38, 823–835. 10.1603/022.038.0336 19508793

[B45] Stadler E. Schoni R. (1991). High sensitivity to sodium in the sugar chemoreceptor of the cherry fruit fly after emergence. Physiol. Entomol. 16, 117–129. 10.1111/j.1365-3032.1991.tb00548.x

[B46] Thiem H. (1954). Wie Ernte Ich Madenfreie Kirschen; Flugblatt K 13, 2. Auflage. Braunschweig: Biologische Bundesanstalt Braunschweig.

[B47] Vogt H. Kaffer T. Just J. Herz A. Féjoz B. Köppler K. (2010). Key biological and ecological characteristics of European cherry fruit fly *Rhagoletis cerasi* with relevance to management, in IOBC wprs WG “Integrated Protection of Fruit Crops.” Joint Meeting of the Sub-Groups “Pome Fruit Arthropods” and “Stone Fruits” (Vico del Gargano).

[B48] Wiesmann R. (1935). Ergebnisse dreijähriger Untersuchungen über die Biologie und Bekämpfung der Kirschfliege *Rhagoletis cerasi* L. in der Schweiz. Anzeiger für Schädlingskunde 11, 97–103. 10.1007/BF02339996

